# An analysis of policy levers used to implement mental health reform in Australia 1992-2012

**DOI:** 10.1186/s12913-015-1142-3

**Published:** 2015-10-24

**Authors:** Francesca C. Grace, Carla S. Meurk, Brian W. Head, Wayne D. Hall, Georgia Carstensen, Meredith G. Harris, Harvey A. Whiteford

**Affiliations:** Sydney Local Health District, Canterbury Hospital, 575 Canterbury Road, Campsie, NSW 2194 Australia; The University of Queensland, School of Public Health, Queensland Centre for Mental Health Research, Locked Bag 500, Sumner Park BC, St Lucia, QLD 4074 Australia; Institute for Social Science Research, The University of Queensland, GPN3 Building, Campbell Road, St Lucia, QLD 4072 Australia; Centre for Youth Substance Abuse Research, The University of Queensland, CYSAR K Floor Mental Health Centre, Royal Brisbane & Women’s Hospital Campus, Herston, QLD 4029 Australia

**Keywords:** Federal Government of Australia, Evidence-based reform, Mental health, Policy levers, Regulation

## Abstract

**Background:**

Over the past two decades, mental health reform in Australia has received unprecedented government attention. This study explored how five policy levers (organisation, regulation, community education, finance and payment) were used by the Australian Federal Government to implement mental health reforms.

**Methods:**

Australian Government publications, including the four mental health plans (published in 1992, 1998, 2003 and 2008) were analysed according to policy levers used to drive reform across five priority areas: [1] human rights and community attitudes; [2] responding to community need; [3] service structures; [4] service quality and effectiveness; and [5] resources and service access.

**Results:**

Policy levers were applied in varying ways; with two or three levers often concurrently used to implement a single initiative or strategy. For example, changes to service structures were achieved using various combinations of all five levers. Attempts to improve service quality and effectiveness were instead made through a single lever-regulation. The use of some levers changed over time, including a move away from prescriptive, legislative use of regulation, towards a greater focus on monitoring service standards and consumer outcomes.

**Conclusions:**

Patterns in the application of policy levers across the National Mental Health Strategy, as identified in this analysis, represent a novel way of conceptualising the history of mental health reform in Australia. An improved understanding of the strategic targeting and appropriate utilisation of policy levers may assist in the delivery and evaluation of evidence-based mental health reform in the future.

## Background

Australia has received international recognition for its mental health policy development [1], particularly in relation to population-based community care services and the recognition of consumer and carer rights [1, 2]. However, some stakeholders have argued that these successes have been overshadowed by a failure to fully implement the promised reforms [2–4]. To help understand the reform process, this study analyses the tools, or policy levers, through which the Federal Government has sought to implement mental health system reform over the past 20 years.

### Australia’s health system and mental health sector

Australia operates a federated system of government with a complex division of responsibilities between Federal and State/Territory Governments. Under a series of intergovernmental agreements, the Federal Government is responsible for the regulation and funding of health services; States and Territories carry primary responsibility for service delivery and management, with some additional regulatory and funding responsibilities [5].

The organisation of tax collection in Australia creates a ‘vertical fiscal imbalance’; the Federal Government collects more tax than it needs to discharge its constitutional responsibilities, while the States and Territories do not collect enough. This imbalance allows the Federal Government to influence State and Territory policies by attaching conditions to its allocation of funds. Thus, while mental health reforms have been progressed by both Federal and State/Territory Governments, the Federal Government has played an increasingly important role in mental health policy.

In this way, mental health reform can be viewed as an example of ‘centralisation’, whereby the Federal Government assumes a more active role in the oversight, public reporting, sanctioning and rewarding of State/Territory policy performance via the conditions it ties to its financial assistance [6]. However, some political theorists have argued that, despite the apparent dominance of the Federal Government, reform can be best understood as an example of ‘decentralised integration’, pointing to evidence of bottom-up or “adaptive” strategizing, such as the adaptation and/or expansion of State-based practice in Federal policy [6, 7].

In the mental health sector, public inpatient and associated community health services are managed and delivered by State/Territory Governments, with shared financial responsibilities between the Federal Government and States/Territories. Responsibility for community-based accommodation and support is also shared, with the Federal Government providing income support to people with mental illness.

Broadly speaking, primary health care, public health and aged care programs do not feature under the State/Territories portfolios. They are delivered and managed by general practitioners (GPs), allied health and other providers [5]. The Federal Government subsidises private general practice, specialist psychiatry or allied health (e.g. psychologist) services through Medicare rebates for fee for service and provides extensive subsidies for psychotropic drugs under the Pharmaceutical Benefits Scheme.

### Mental health policy in Australia 1992–2012

The analysis we present here focuses exclusively on Federal Government level reforms that began in 1992.

The inception of a National Mental Health Strategy (NMHS) and the first five-year National Mental Health Plan in 1992 marked an important turning point for mental health in Australia [8, 9]. The subsequent 20 years of mental health reform saw the publication of further five-yearly Government Plans, each of which outlined priority areas for investment and reform. The first Plan (1993–98) primarily addressed problems in community mental health care, arising from thirty years of deinstitutionalisation [10].

The second Plan (1998–2003) expanded the policy focus beyond severe mental illness to more common mild and moderately severe forms of mental disorder [11]. Priority was given to the role of GPs and primary health practitioners, who provided most of the treatment for these common mental disorders, with an emphasis on prevention and early intervention [11].

The third Plan (2003–08) [12] gave priority to workforce enhancement and better integration across all systems of care, including legal, emergency and substance abuse services. Critics suggested that the third Plan spread the available resources too thinly by attempting to address all problems raised in the first and second Plans, in the absence of additional Federal Government funding or specific guidance on actionable and measurable outcomes [13].

The fourth Plan (2008–13) sought to strengthen workforce agreements, accreditation and reporting standards, and to deliver resource packages and outcome targets tailored to particular minority groups, such as Indigenous Australians [14]. The fourth Plan did not receive any significant resource allocation however, as it had been superceded by a whole of government National Action Plan on Mental Health [15].

The four National Mental Health Plans were endorsed at the level of the Federal Health Minister whereas the five-year National Action Plan on Mental Health was endorsed in 2006 by the Council of Australian Governments (COAG), at the level of the Prime Minister, State Premiers and Territory Chief Ministers [16]. The COAG plan detailed nine individual implementation plans, accompanied by key reform strategies and outcome measures, and promised a $1.8 billion Federal Government investment in mental health [16].

The next major Federal Government investment was a $2.2 billion injection of funding for mental health released as part of the 2011–12 Budget [17]. This promoted a shift towards funding early intervention and prevention, with a particular focus on delivering innovative youth support services [17].

### Conceptual framework

This paper employs Kingdon’s conceptualisation of the policy process as a general operating framework [18]. Under this framework, the policy process is seen as comprising of three streams: problems, policies and politics [18]. Each stream contains its own processes and actors, operating independently, with its own set of rules and dynamics [18]. At certain times, when all three streams are coupled by “political entrepreneurs”, a “policy window” is opened, creating an opportunity for policy change [18]. Although Kingdon’s model was designed from studying the US political system, it has been applied to health reform (including mental health reform) in Australia [19].

Whilst acknowledging the importance of all phases of the agenda setting process, including the role of advocacy, this paper focuses on mapping policy problems to solutions, specifically, the use of tools, or policy levers, applied under the National Mental Health Strategy (NMHS).

### Policy levers—the “control knobs” of the health system

Governments have a restricted range of tools, or policy levers, at their disposal to implement broad scale health reforms [20–23]. Policy levers are instruments that can be adjusted by governments to achieve system-wide change [20–23]. A variety of policy levers can be applied by governments to deliver health service reform; with the choice of lever being influenced by the political climate, as well as constitutional and legal restrictions on government authority [23].

There is no universally accepted typology of policy levers, with various researchers and disciplines advancing different classifications [22]. We have used the typology proposed by Roberts *et al.* [21], the World Bank Institute and others, to identify and classify health sector policy levers in terms of: organisation, regulation, community education, finance and payment (Table 1).

Roberts *et al.* [21] argues that a policy lever should: [1] represent a discrete area of health system structure or function that is amenable to adjustment by Government action, and [2] play a causal role in health system performance. Policy levers are distinguished from causal factors affecting health system performance that either lie outside the control of the health sector and/or are not easily affected by public policy e.g. war, economic growth, climate and cultural norms [21, 24].

**Table 1:** Definitions and examples of five policy levers, as applicable to a health system context (adapted from Roberts *et al.* [21])

Lever	Definition	Example
Organisation	Macro-level changes in location, magnitude, co-ordination and diversity of human and physical capital	Establishment of local hospital networks
Regulation	Enforced changes in behaviour	Service standards for healthcare professionals
Community Education	Spreading of information to influence changes in behaviour	Mass media health education campaigns
Finance	Revenue generation and allocation/distribution of funds	Subsidies for private health insurance
Payment	System of incentives for health providers	Activity-based hospital funding

## Methods

The present analysis focuses on the implementation of key initiatives from the commencement of the NMHS in 1992, until 2012, in terms of the five policy levers defined by the World Bank Institute and others [21] (organisation, regulation, community education, finance and payment).

Particular emphasis is given to the following four Australian Federal Government publications, which were the most influential in terms of announcing and directing key policy and service delivery initiatives [10, 11, 16, 17].

1. National Mental Health Plan 1992
2. Second National Mental Health Plan 1998
3. COAG National Action Plan on Mental Health 2006–2011
4. Federal Health Budget: National Mental Health Reform 2011–12

Formal Government funded evaluations of each of major policy shift were also referenced where they provide further elaboration on the levers used [13, 25–29].

Information about policy and reform initiatives was extracted from the reference documents. Strategies and deliverables identified in each of the Plans were summarised and organised according to:

1. key priority reform areas [i] human rights and community attitudes; [ii] responding to community need; [iii] service structures; [iv] service quality and effectiveness; and [v] resources and service access [19];
2. policy lever (s)used in its implementation (as per below definitions) [i] organisation; [ii] regulation; [iii] community education; [iv] finance and [v] payment [21]

### Organisation

Organisation refers to macro-level changes in the location, magnitude and diversity of capital and human resources provided across primary, secondary and tertiary care facilities [21]. It also includes the degree of vertical integration between these types of services [21]. Policy directives that primarily seek to influence the degree of integration and/or centralisation or to influence the type(s) of ownership of health facilities (i.e. private, public, not-for-profit) may be classified under the organisation lever.

### Regulation

Regulation pertains to the use of government power to enforce changes in behaviour [21]. The purpose of regulation is commonly to correct for market failures, to protect consumers by ensuring quality and safety standards are met, and to achieve non-market goals [21]. Effective regulation requires a strong technical competence and resource base, and is facilitated by the existence of consensus and support across both public and political sectors [21].

### Community education

Originally described by Roberts *et al.* [21] as ‘behaviour’, community education involves disseminating information to influence the behaviour of individuals and organisations [21]. Successful application requires information to be tailored to the needs and values of a target group [30]. Focus areas include preventive health measures, lifestyle choices, help-seeking and treatment compliance [30]. Community education often takes place via mass media channels and can involve explicit persuasion and marketing [21].

### Finance

Finance relates to ways the mental health sector can generate revenue. Finance levers control the overall allocation and distribution of funding across Government (Federal, State/Territory and Local), private and not-for-profit sectors [21]. Finance influences the degree of control and responsibility accorded to each type of service provider, as well as the overall sector’s capacity and reach [21].

### Payment

Payment is a system of fiscal incentives used to control the quantity and quality of services provided by health professionals [21]. This lever describes the choice of a unit or basis for professional payment (e.g. consultation or treatment plan) and the rate of pay per unit [21]. The choice of payment system is dependent upon the organisation and mode of delivery of a service, including the number of providers and the alternatives available [21]. It also reflects the importance placed on different types of service provision.

## Results

Table 2 summarises each of the main reform initiatives of the NMHS 1992–2012, according to Plan and then policy lever. Reform initiatives that represented an allocation of resources towards the augmentation or enhancement of existing service models, rather than new models of care, are italicised. Each initiative is also categorised according to the key priority area to which it relates. Elaboration of the use of levers relative to each priority area is provided in the subsequent paragraphs.

**Table 2:** Application of policy levers to reform priority areas across the NMHS 1992-2012

	Organisation	Regulation	Community education	Finance	Payment
First Plan (1993-98)	*SS*	*HR&CA*	*HR&CA*	*SQ&E*	
	Mainstream mental health service management	Review consumer rights and responsibilities	National Community Awareness Program	Separate budget for mental health	
	*SS*	*HR&CA*		*R&SA*	
	Shift acute beds to general hospitals	Review mental health and anti-discrimination legislation		*Increase recurrent mental health spending*	
	*RCN*	*SS*		*R&SA*	
	Formalise consumer/carer consultation	Remove cross-border transfer anomalies		*Increase community-based and general hospital funding*	
	*SS*	*SQ&E*		*R&SA*	
	Case Management system	National Mental Health Information strategy and National Minimum Dataset		Review Medicare Agreements	
	*SS*	*SQ&E*			
	*Increase ambulatory workforce*	Independent evaluation steering committee and National Mental Health Commission			
		*SQ&E*			
		Annual National Mental Health Reports			
		*SS*			
		Review interagency protocols to support continuity of care			
Second Plan (1998-2003)	*SS*	*SQ&E*	*HR&CA*	*SS*	*SS*
	Shift acute beds to general hospitals	National Standards for Mental Health Services	Review media portrayal of mental illness	Redirect funds released from deinstitutionalisation towards community-based services	New funding models to improve referral links between primary and secondary providers
	*RCN*	*SS*	*HR&CA*		*SS*
	Formalise consumer/carer consultation	Review interagency protocols to support continuity of care	Mental health education in schools		Review Medicare Benefits Scheme (MBS) items and introduce new MBS items to support referral between health practitioners
	*SS*	*SQ&E*	*HR&CA*	*R&SA*	*R&SA*
	*Grow 24-h staffed community based residential services*	New outcome measures	Mental health training for health professionals, in particular frontline workers	Review allocations under the general health budget for Federal and State/Territory Governments	Specialised funding models for rural and regional populations
	*R&SA*				
	*Increase treatment rates for mild and moderate mental illness*				
	*R&SA*				
	*Improve mental health curricula for Indigenous health workforce*				
	*R&SA*				
	Specialist youth early intervention centres (e.g. Headspace, Early Psychosis Prevention and Intervention Centres (EPPIC)				
	*R&SA*				
	Specialised service models for special needs populations				
Council of Australian Governments (COAG) Action Plan (2006)	*R&SA*	*SQ&E*	R&SA		*R&SA*
	24-h 7 day mental health telephone service	COAG Mental Health Groups in each jurisdiction	Review mental health content in tertiary health degrees		Mental Health Nurse Incentive Program
	*R&SA*	*SQ&E*	*HR&CA*		*R&SA*
	*Increase web-based mental health resources*	Publish official Progress reports annually	Mental health training for health professionals, in particular frontline workers		Flexible employment schemes and funding models for rural and regional areas
	*SS*				*SS*
	Consolidate youth mentoring and early intervention programs into a single program				Review existing MBS items and introduce new MBS items to support referral between health practitioners
	*R&SA*				
	New youth early intervention projects				
	*RCN*				
	Family Mental Health Support Service				
	*RCN*				
	*Increase mental health respite services for carers*				
	*R&SA*				
	Step-up and step-down community facilities				
	*ISS*				
	Community Coordinators				
	*SS*				
	*Increase day-to-day living support programs and Personal Support program*				
	*RCN*				
	*Increase Personal Helpers and Mentors service*				
	SS				
	Disability Employment Services group				
	*R&SA*				
	*Increase supported places in University mental health degrees, particularly to Indigenous students*				
	*R&SA*				
	*Increase Indigenous health workforce*				
	*R&SA*				
	*Additional funding to drug and alcohol service providers*				
2011-12 Budget	*SS*	*SQ&E*		*R&SA*	*R&SA*
	Care Facilitator scheme	National Mental Health Commission		National Funding Pool for States/Territories	Reduce Medicare rebate for General Practitioner mental health plans and introduce tiered rebate system
	*RCN*	*SQ&E*			*R&SA*
	*Additional Personal Helpers and Mentors and places in Family Mental Health Support Services*	Fund research into improving data sets and performance accountability			Reduce annual number of rebated consultations with allied psychological services
	*R&SA*	*SQ&E*			*R&SA*
	E-health portal for mental health	Annual Report Card on mental health reform			*Expand Access to Allied Psychological Services scheme*
	*R&SA*	*SQ&E*			*SS*
	*Additional Headspace and EPPIC sites*	Australian Early Development Index tool and an expert group in child mental health			Review wage-subsidy schemes and Disability Support Pension
	*R&SA*	*SS*			
	Routine Health and Wellbeing checks for 3 year olds	National Partnership Agreements and new Service Planning Framework			
	*RCN*				
	National Mental Health Consumer Body				

Key Priority Areas: Human Rights and Community Attitudes (HR&CA), Responding to Community Need (RCN), Service Structures (SS), Service Quality and Effectiveness (SQ&E) and Resources and Service Access (R&SA)

### Human rights and community attitudes

Human rights and community attitudes, including mental health literacy, were identified as priority areas throughout the NMHS, but the approaches used and target audiences varied over time. Regulation was initially used to improve awareness, acceptance and tolerance of persons with mental disorders. This was informed by a systematic review of mental health and anti-discrimination legislation [25]. There was also a commitment to improving the respect afforded to consumers and carers, in accordance with the Mental Health Statement of Rights and Responsibilities 1991 [31].

Implementation subsequently narrowed to a focus on community education [7, 25]. A National Community Awareness Program was introduced to improve community mental health literacy and reduce stigmatisation of mental illness [19]. Mental health literacy refers to public knowledge and beliefs about mental illnesses and their treatment [25, 27]. Improvements to community health literacy can assist in the early detection, management and prevention of mental disorders [27]. A national media strategy for mental health, Mindframe, was also developed, in response to a review of the media’s portrayal of mental illness [32].

Education reform efforts also focused on frontline providers across both the health and broader service sectors [25]. This was partly in response to consumers’ reports of health practitioners’ disempowering and stigmatising attitudes, as identified in the 1997 National Survey of Mental Health and Wellbeing [25, 26]. Efforts to reform practitioner attitudes included the provision of mental health training in tertiary education and professional development, and the distribution of literature [26].

### Responding to community need

The 1997 National Survey of Mental Health and Wellbeing demonstrated that mild and moderately severe anxiety and depressive disorders were the most prevalent mental disorders, and the Australian Burden of Disease Study found they made the most substantial contribution to disability in Australia [33, 34]. Organisation was used to facilitate increased access to treatment for persons with these disorders through primary care, rather than specialist psychiatric services. This was in response to survey findings that care for common mental disorders was predominately delivered in primary care by GPs [26, 33] The organisational shift towards mental healthcare delivery in primary healthcare settings heralded a new set of responsibilities for the Federal Government [26]^.^ The Joint Consultative Committee in Psychiatry (1997) proposed that primary care psychiatry “could be regarded as the last frontier of mainstreaming” [33].

Community education efforts aimed to complement the focus on early intervention by promoting awareness of risk factors for mental illness [16]. Youth Pathways projects were introduced under the COAG Plan to reduce school dropout rates related to mental illness, and the National Suicide Prevention Strategy was expanded [16]. Non-Government Organisations (NGO) also offered support and education to parents and carers of children with mental illness. This included assistance for early detection of symptoms, and referrals to appropriate sources of help [16]. Additional Federal Government funding (finance) was set aside for the provision of new community service platforms for young people including Headspace, Youth Pathways and Early Psychosis Prevention and Intervention Centre (EPPIC) centres [17]^.^ These services promoted the early detection and treatment of mental illness via a further decentralisation of mental health care [17].

Finally, organisational changes were introduced to promote increased consumer and carer participation in both private and public sector organisations [17, 29]. A National Mental Health Consumer Body was established to embed consumer and carer experience within policymaking, program development and implementation [17].

### Service structures

A third major focus area was to change the structure of services, including the mainstreaming of mental health services [17]. Changes that were implemented primarily at the organisational level included the closure of beds accepting acute admissions in stand-alone institutions, and their relocation to general hospitals [35]. This was complemented by the integration of mental health service management into area-based general health services [25, 26]. Funds released after deinstitutionalisation (finance) were intended for community-based services at both the Federal and State/Territory level [25].

A variety of approaches were used to facilitate improved coordination of care for patients, across service providers and jurisdictions [25, 26]. Regulation, in the form of legislative amendments, established joint protocols between service providers across service sectors to support coordination of care [25, 26]. Organisation was used to facilitate the introduction of case managers/facilitators, responsible for individual patient care across service providers, and to increase step-down facilities [16]. Payment, through Better Outcomes in Mental Health Care and Better Access to Psychiatrists, Psychologists and General Practitioners through the Medicare Benefits Scheme (MBS), was used to improve referrals between primary and secondary providers [26]. These incentive programs allocated a specific MBS provider payment to coordinate and plan services [26].

The strategic use of finance to promote service structure changes increased during the later half of the NMHS, with targeted funding allocated to areas of perceived community need [26]. This included an unprecedented increase in Federal Government funding to NGOs for the provision of day-to-day support and employment/vocational services for people with mental illness [26]. There was also increased Federal Government funding for NGO-orchestrated respite services for carers [17]. Alongside finance, community education was used to better inform frontline staff regarding the availability of employment and social support services, and to help facilitate their utilisation [27].

### Service quality and effectiveness

The goal of improving service quality and standards in mental health care was addressed primarily through regulation. Initially, the focus was on making changes to national service protocols to better reflect United Nations standards [26].

As reform progressed, the focus of regulation centred on tightening accountability for Federal (and State/Territory) Governments in delivering better outcomes [25, 26]. A National Minimum Dataset was developed, and approximately two-thirds of organisations were engaged in strategic and routine outcome monitoring by the end of the second Plan [28]. The Australian Health Minister’s Advisory Committee appointed an Evaluation Steering Committee whose role was to independently evaluate the NMHS [28]. Under the 2011–12 Budget, a new National Mental Health Commission was established to deliver an Annual Report Card on Mental Health and Suicide Prevention to Parliament, through the Prime Minister [17].

### Resources and service access

Continuity-of-care initiatives were also supported through regulation, to improve service access. This included a review of other Government agency guidelines and, where necessary, the establishment of joint protocols and formal agreements [25]. To facilitate the transfer of patients across jurisdictions, administrative and legislative requirements were simplified and cross-border anomalies identified [25]. National Partnership Agreements on Mental Health and a new National Service Planning Framework were introduced to promote a nationally consistent assessment process, address service gaps and promote multiagency support [16].

Finance was applied alongside regulation, to establish clearer roles and bilateral funding arrangements between Federal and State/Territory departments, under the Medicare Agreements Act 1993–1998 [28]. For the first time, mental health was formally quarantined from other health services in the Medicare Agreements [28]. A separate budget was established for mental health and there was an increase in recurrent mental health spending [28].

Beginning with the second Plan, earmarked funding (finance) was targeted towards mental health care for rural/remote residents and Indigenous Australians [17]. New payment models were also developed under the Divisions of Practice scheme 25. These incorporated flexible employment and incentive schemes to improve access to treatment services in rural and remote areas [26]. The Mental Health Nurse Incentive Program was introduced in July 2007, featuring new funding and targeted incentives to promote their involvement in patients’ existing care programs, alongside GPs and/or psychologists [27]. This combined use of payment and finance sought to increase the health workforce, and thereby improve service access [26, 27].

Organisational change increased access to alternative service delivery methods for rural and remote communities [26]. This included telephone services and an e-mental health portal, to facilitate easy self-directed access to online mental health resources and treatment options [27]. A 24-h 7 days a week mental health telephone service was linked to the National Health Call Centre Network [29]. These reforms were accompanied by dedicated funding (finance) for NGOs for the provision of these services. Supportive payment models were introduced via additional MBS items such as tele-psychiatry [26, 27].

## Discussion

The present, classificatory analysis offers novel insight into how policy levers have been used by government to achieve mental health reform. By describing NMHS initiatives in accordance with the policy levers employed in their realisation, this work provides a framework for further evaluation of their relative successes and failures, to inform future health policy development in Australia and similarly organised liberal democracies.

Public administration is not a politically neutral nor purely rational exercise. Rather, it requires negotiation and compromise between multiple political actors [36]. The choice of policy levers will be affected by values and political ideologies, and will reflect the outcome of negotiations between different levels of government, factions within ruling parties, and policy entrepreneurs who advance specific policy ‘solutions’ as part of their advocacy [18]. Policy entrepreneurs have been particularly important with respect to the specific areas of mental health (e.g. human rights and community attitudes, responding to community need etc.) given priority in each wave of reform, and around which our analysis of levers is structured [18, 19].

These dynamics do not, however, undermine the value of bracketing out such political processes, to focus on the classification and application of different policy mechanisms, offering a schema for their future evaluation.

All five policy levers in the World Bank Institute typology [21] were used to achieve reform under the NMHS. Often, two or three levers were used concurrently to implement a single initiative or strategy. The emphasis given to different kinds of levers varied over time, in response to changing political and other circumstances.

Although the types of levers used varied across the major four policy shifts, several key patterns were evident. Changes to service structures were primarily achieved through organisational change in combination with community education, and changes in payment, regulation or finance.

The use of payment as a policy lever increased over time. This reflects increasing Federal Government intent and capacity to control health policy and service reform through standardised payment for services. Increased use of formal incentive structures and outcomes-based funding may have also been in response to criticisms that service providers had not been held accountable under earlier reforms [19].

The use of regulation also changed over the course of the reforms. It moved from a prescriptive, legislative approach, toward a greater focus on improving service standards and consumer outcomes. Early examples of bold, entrepreneurial strategies, such as formal recognition of consumer rights and responsibilities, were later superseded by adaptive strategizing and decentralised integration, with the Federal Government recognising autonomy and diversity of States and Territories in lieu of jurisdictional uniformity [6, 7]. For example, dissolution of cross-border anomalies was an important focus of the first and second Plans. Conversely, under the COAG Plan and 2011–12 Budget, routine monitoring against key national performance indicators (adopted based on a Victorian model), tended towards recognition of likely diversity in the degree and means of achievement between States and Territories [6]. This changing use of regulation may reflect ongoing negotiation and bargaining between Federal and State/Territory authorities.

Many of the regulatory changes perceived as necessary were formally adopted under the first and second Plans, with attention subsequently diverted to the practical implementation and realisation of these changes. This shift may also indicate increasing recognition of the capacity for community education, rather than regulation, to influence public attitudes [28].

Financial changes also appeared to accompany most, if not all, uses of the other four levers. This is perhaps unsurprising, because government funding is required to support and sustain the implementation of any new initiative and is therefore often a necessary consideration for policy change. Over the 20-year period, there were very few examples of implementation that exclusively used finance. Notable exceptions included the introduction of a separate mental health budget, increases to overall portfolio expenditure and the introduction of competitive funding allocations for States/Territories [24]. This increased tendency to use finance in conjunction with other policy levers is perhaps a reflection of the Federal Government’s desire to have greater control over the use of its funds.

In later years, NGOs such as beyondblue, became increasingly responsible for implementing community education and improving research and mental health awareness [29]. This shift was accompanied by a simultaneous decrease in the Government’s direct use of community education. This appears to reflect a growing perception within government that NGO services can best meet the needs of mental health consumers; potentially a result of successful lobbying by the Mental Health Association and others in favour of greater NGO involvement [37].

In addition to contributing to mental health policy studies, our analysis serves a further purpose, albeit one directed at mental health services researchers rather than policy analysts or political actors. Researchers are increasingly expected to produce and then ‘translate’ findings into policies and practices that achieve outcomes with demonstrable social benefit. Yet, for many health researchers, the political institutions with which they must engage in order to achieve such research translation can be mysterious and ‘irrational’. In highlighting some of the structural elements underpinning policy implementation, we aim to speak to these knowledge producers and demystify this process as one that is about more than politics.

### Limitations and future research directions

The five levers identified by the World Bank as most relevant for application to the health sector were used to analyse implementation strategies across the four major policy shifts. It is recognised that these five policy levers may not represent a comprehensive list of policy mechanisms used in the mental health sector. The policy and reform initiatives featured in each of the reference documents were extracted and ascribed to one of the five pre-defined categories. It is possible that additional lever(s), not included within the World Bank typology, may have been applied. Future research could build upon the present analysis by exploring the iterative addition or substitution of levers from alternative typologies. This process may allow for the discovery of any additional levers relevant to health policy reform, if required.

This research presents an important first step towards evaluating mental health reform under the NMHS. Future research should build upon the efforts of the present analysis by appraising the success or failure of levers in achieving their intended outcomes. This is a complex undertaking due to the multidimensional character of success and failure, political influences on evaluations and media publications, and empirical limitations [38, 39].

Our research did not include a specific analysis of the political dimensions and negotiations influencing policy lever selection, however we do not think its omission undermines the value of the current approach. By identifying patterns in the application of policy levers to priority areas, our classificatory analysis can help shine a light on assumptions and political ideologies influencing the choice of tools to implement reforms. The increasing reliance on financial drivers and devolution of responsibility to the non-government sector, reflecting neoliberal modes of governance, is a pertinent case in point and warrants further critical scrutiny.

## Conclusions

To the best of our knowledge, this paper represents the first analysis of policy lever use to achieve mental health reform in Australia. It represents a novel exploration of the relevance of the current World Bank framework to this area of mental health, with implications for policy-makers and researchers. To date, no comparable publications have attempted to characterise mental health reform efforts in terms of the choice and application of policy levers available to government. An improved understanding of the strategic application of the levers available to Government may assist the future implementation and evaluation of evidence-based policy.

## Abbreviations

COAG: Council of Australian Governments
EPPIC: Early Psychosis Prevention and Intervention Centre
MBS: Medicare Benefits Scheme
NGO: Non-government organisation
NMHS: National Mental Health Strategy
